# Gender Disparity in Nutritional Status and Education Among Children Aged Seven to 12 Years in Jasra Block, Prayagraj, Uttar Pradesh, India: A Community-Based Cross-Sectional Study

**DOI:** 10.7759/cureus.111372

**Published:** 2026-06-23

**Authors:** Srishti Chauhan, Khurshid Parveen, Ashutosh Kumar

**Affiliations:** 1 Community Medicine, Sarojini Naidu Medical College, Agra, IND; 2 Community Medicine, Moti Lal Nehru Medical College, Allahabad, IND

**Keywords:** child nutrition disorders, double burden malnutrition, gender disparity, gender equity, height-for-age, nutritional status, prayagraj, rural population, school-age children, stunting

## Abstract

Background: Malnutrition and educational disadvantage among children remain important public health concerns in rural India, where intra-household food allocation, healthcare-seeking, and educational opportunities may differ between boys and girls. This study assessed gender disparities in nutritional status and education among children aged seven to 12 years in a rural community of Prayagraj, Uttar Pradesh.

Materials and methods: A community-based cross-sectional study was conducted among 486 children aged seven to 12 years (243 boys and 243 girls) in Jasra block, Prayagraj, using consecutive house-to-house enrolment. Data were collected through a pre-designed, pre-tested schedule administered to mothers/caregivers and through direct anthropometric examination. Nutritional status was assessed using height-for-age and body mass index-for-age categories. Educational status, school status, school type, and sociodemographic variables were recorded. Associations with gender were assessed using the chi-square test. Binomial logistic regression was performed with educational and nutritional status as outcomes and gender as the exposure. A p<0.05 was considered significant.

Results: Of 486 children, 405 (83.33%) were ever enrolled, and 81 (16.67%) were never enrolled. Never enrolment was significantly higher among girls (54, 22.22%) than boys (27, 11.11%; p=0.001). Height-for-age was significantly associated with gender (p=0.008); moderate stunting (28 {11.52%} girls vs. 16 {6.58%} boys) and severe stunting (36 {14.81%} girls vs. 29 {11.93%} boys) were more frequent among girls. BMI-for-age did not show a statistically significant association with gender (p=0.193); although moderate thinness (44 {18.11%} girls vs. 35 {14.40%} boys) and severe thinness (49 {20.16%} girls vs. 39 {16.05%} boys) were proportionately higher among girls, this represents a non-significant observed trend. With gender as the exposure, girls had higher odds of never enrolment (OR 2.29; 95% CI: 1.38-3.77; p=0.001) and of moderate-or-severe stunting (OR: 1.57; 95% CI: 1.02-2.42; p=0.040), whereas the association with moderate-or-severe thinness was not significant (OR: 1.42; 95% CI: 0.97-2.06; p=0.070).

Conclusion: Gender disparity was evident in educational access and in chronic undernutrition (stunting) among children aged seven to 12 years in Jasra block, with girls disadvantaged on both. BMI-based thinness was higher among girls but did not reach statistical significance and should be interpreted as a trend. The findings support gender-sensitive school health services, community-based nutrition screening, household counseling on equitable food distribution, and active tracking of girls who are never enrolled or have dropped out.

## Introduction

Malnutrition among children remains one of the most persistent public health challenges in India, with long-term consequences for growth, immunity, learning capacity, productivity, and intergenerational health. Inadequate intake of calories, protein, and micronutrients during childhood can manifest as underweight, stunting, wasting, or other forms of nutritional deficiency, while overnutrition may coexist as part of the broader double burden of malnutrition [1]. School-age children represent a particularly important group, as this period is marked by continued physical growth, cognitive development, social learning, and educational progression. Nutritional deprivation during these years can influence school participation, attention span, educational attainment, and future human capital.

Gender is an important social determinant of child health and nutrition in India. Beyond biological vulnerability, nutritional outcomes are shaped by household-level food allocation, caregiving priorities, healthcare-seeking behavior, parental education, socioeconomic status, and prevailing gender norms. Hathi et al. described intra-household food allocation as a form of gender discrimination, noting that girls and women may receive less or poorer-quality food and often eat after men [2]. Conceptually, therefore, nutrition is not only a biological state but also a marker of the value assigned to different household members; gender disparity in nutrition is expected to be mediated by these social processes rather than determined by sex alone.

Evidence from India is heterogeneous. In rural Bijapur, Karnataka, Jawaregowda and Angadi reported higher undernutrition among boys [3], whereas Tripathi et al., studying children aged seven to 12 years in rural Allahabad, found severe underweight and certain stunting patterns higher among girls [4]. Yesmin and Baruah reported greater female disadvantage in urban Assam [5]. Regional studies from Uttar Pradesh, including those by Pal and Gautam from Varanasi and by Kumari et al. from Prayagraj, document a substantial and persistent burden of childhood malnutrition [6,7]. Education is closely linked with nutrition and gender equity, and evidence from rural Prayagraj indicates that adolescent girls experience poor dietary diversity and undernutrition alongside educational and health disadvantage [8].

Despite this literature, several gaps remain. Many studies focus on children aged under five years or adolescents, with fewer examining the seven to 12-year age group. Nutritional and educational disparities are often studied in isolation, and there is limited recent community-level evidence from rural Prayagraj assessing both domains using primary household-level data. This gap is important because school-age children are at a critical stage where nutritional disadvantage and educational exclusion can reinforce one another. The present study was therefore conducted in the Jasra block, a rural community in Prayagraj, among children aged seven to 12 years, to assess gender disparities in nutritional status and education and to identify factors associated with these disparities.

## Materials and methods

Study design and setting

This was a community-based cross-sectional observational study conducted in Jasra block, Prayagraj district, Uttar Pradesh, India, over a two-month period (April 2024-May 2024). Jasra block was selected as the defined rural study area in view of prior indications of gender gaps in child health indicators and to allow feasible house-to-house data collection, direct anthropometric assessment, and uniform field supervision.

Study population and eligibility

Children aged seven to 12 years residing in the study area whose mothers/caregivers were available for interview and provided consent were eligible for inclusion in this study. Children with any serious medical condition that could affect anthropometric assessment or interfere with participation were excluded.

Sample size

The sample size was calculated for the comparison of gender disparity in severe malnutrition among children aged seven to 12 years. The prevalence of severe underweight among boys and girls was taken from the study by Tripathi et al., conducted among school-going children in a rural community of Allahabad, Uttar Pradesh, where severe underweight was reported as 8.8% among boys and 17.3% among girls [4]. Using these proportions, with a 95% confidence level (CI) and 80% power, the sample size was calculated using the below formula for comparison of two proportions.

$n = \frac{(Z_{\alpha/2} + Z_{\beta})^2 \left[ P_1(1 - P_1) + P_2(1 - P_2) \right]}{(P_1 - P_2)^2}$

Here, P1 was the prevalence of severe malnutrition among boys, P2 was the prevalence of severe malnutrition among girls, Z_α/2_ was 1.96, and Zβ was 0.84. The calculated sample size was approximately 243 children per gender group, giving a total sample size of 486 children. The final study sample included 243 boys and 243 girls.

Sampling method

A community-based consecutive sampling method was used. House-to-house visits were conducted across the selected rural community, and all eligible children whose caregivers were available were approached consecutively and enrolled after obtaining consent until the required sample size was reached, maintaining equal representation of boys and girls.

Data collection and anthropometry

Data were collected through house-to-house visits using a pre-designed, pre-tested schedule covering age, gender, religion, family type, number of family members, monthly family income, B.G. Prasad socioeconomic class, educational status, school status, type of school, and clinical features. Data were collected by trained personnel who underwent prior orientation in interview techniques and standardized anthropometric measurements to minimize inter-observer variation. Weight was measured in kilograms using a standard weighing scale with the child barefoot in light clothing; height was measured in centimeters using a measuring tape, with the child standing upright, heels together, and the head in the Frankfurt plane. Body mass index (BMI) was calculated as weight (kg)/height (m²).

Nutritional, educational, and socioeconomic assessment

Nutritional status was assessed using height-for-age (normal, mild, moderate, and severe stunting) and BMI-for-age (normal, moderate thinness, and severe thinness) categories derived from the WHO 2007 growth reference for five to 19 years [9]. Educational status was categorized as ever enrolled or never enrolled. School status was recorded as currently studying, dropout, or never enrolled; current-schooling status (studying vs. dropout) among ever-enrolled children was recorded where reported by the caregiver. The type of school was categorized as government, private, or never admitted. Socioeconomic status was classified using the B.G. Prasad scale, updated to 2024, with the applicable All India Consumer Price Index (industrial workers) linking factor, based on per-capita monthly income.

Statistical analysis

Categorical variables were summarized as frequencies and percentages, and continuous variables as mean±standard deviation or median. Associations between gender and categorical variables were assessed using the chi-square test. Binomial logistic regression was used to estimate the odds of adverse educational and nutritional outcomes, with these outcomes as dependent variables and gender (female vs. male) as the exposure. A p<0.05 was considered statistically significant.

## Results

The baseline sociodemographic and anthropometric characteristics of the study participants according to gender are presented in Table 1. The study included an equal number of males and females, with 243 participants in each group. The mean age was slightly higher among females than males (9.34±1.74 years vs. 9.18±1.67 years). Most children were Hindu and belonged to nuclear families. The largest proportion of participants belonged to B.G. Prasad's socioeconomic Class II. Mean weight was higher among males, whereas mean height was comparable between males and females. Body mass index (BMI)-based thinness was more frequent among females, with 44 (18.11%) having moderate thinness and 49 (20.16%) having severe thinness, compared with 35 (14.40%) and 39 (16.05%) among males, respectively.

**Table 1:** Baseline sociodemographic and anthropometric characteristics of study participants according to gender. Values are expressed as frequency with percentage unless otherwise stated. Age is expressed in years, weight in kilograms, height in centimeters, and socioeconomic status as monthly family income in Indian rupees. Body mass index is expressed in kg/m².

Variables	Category/measure	Male (n=243)	Female (n=243)	Total (n=486)
Age (years), n (%)	7 years	46 (18.93)	50 (20.58)	96 (19.75)
	8 years	62 (25.51)	44 (18.11)	106 (21.81)
	9 years	23 (9.47)	33 (13.58)	56 (11.52)
	10 years	60 (24.69)	42 (17.28)	102 (20.99)
	11 years	18 (7.41)	38 (15.64)	56 (11.52)
	12 years	34 (13.99)	36 (14.81)	70 (14.40)
	Mean±standard deviation	9.18±1.67	9.34±1.74	-
Religion, n (%)	Hindu	227 (93.42)	219 (90.12)	446 (91.77)
	Muslim	16 (6.58)	24 (9.88)	40 (8.23)
Type of family, n (%)	Nuclear	167 (68.72)	152 (62.55)	319 (65.64)
	Joint	76 (31.28)	91 (37.45)	167 (34.36)
Family members	Median	5	5	-
	Mean±standard deviation	5.51±2.98	5.57±2.83	-
Socioeconomic status, monthly family income in Indian rupees	Median income	3000	4000	-
	Mean±standard deviation	5364.61±5586.77	5108.23±3919.66	-
B.G. Prasad socioeconomic class	Class I	38 (15.64)	35 (14.40)	73 (15.02)
	Class II	87 (35.80)	86 (35.39)	173 (35.60)
	Class III	69 (28.40)	66 (27.16)	135 (27.78)
	Class IV	42 (17.28)	48 (19.75)	90 (18.52)
	Class V	7 (2.88)	8 (3.29)	15 (3.09)
Weight (kg)	Mean±standard deviation	23.70±6.02	22.54±5.48	-
Height (cm)	Mean±standard deviation	126.48±11.04	126.88±12.06	-
Body mass index category	Normal	169 (69.55)	150 (61.73)	319 (65.64)
	Moderate thinness	35 (14.40)	44 (18.11)	79 (16.26)
	Severe thinness	39 (16.05)	49 (20.16)	88 (18.11)
Body mass index by category	Normal, mean±standard deviation	16.02±2.39	15.25±1.71	-
	Moderate thinness, mean±standard deviation	13.28±0.55	13.02±0.66	-
	Severe thinness, mean±standard deviation	10.58±1.79	10.73±0.92	-

Educational status, school type, and height-for-age were significantly associated with gender, whereas BMI-for-age was not associated (Table 2). Never enrolment was significantly higher among females (54, 22.22%) than males (27, 11.11%; p=0.001). The same 81 never-enrolled children (27 males, 54 females) are reflected consistently under educational status, school status (never enrolled), and school type (never admitted). For school type, the great majority of both sexes depended on government schooling (boys 205, girls 179), with few in private schools (males 11, females nine). The gender difference was driven mainly by the higher proportion of never-admitted females. Height-for-age was significantly associated with gender (p=0.008) - moderate stunting (28 {11.52%} females vs. 16 {6.58%} males) and severe stunting (36 {14.81%} females vs. 29 {11.93%} males) were more common among females. BMI-for-age was not significantly associated with gender (p=0.193); moderate thinness (44 {18.11%} females vs. 35 {14.40%} males) and severe thinness (49 {20.16%} females vs. 39 {16.05%} males) were proportionately higher among females, but this difference represents a non-significant observed trend rather than a confirmed association.

**Table 2:** Association of educational and nutritional status with gender. Percentages are calculated column-wise within each gender group. The chi-square test was applied to assess the association between gender and categorical variables. A p<0.05 was considered statistically significant.

Variables	Category	Male, n (%)	Female, n (%)	p-Value
Educational status	Ever enrolled	216 (88.89)	189 (77.78)	0.001
	Never enrolled	27 (11.11)	54 (22.22)	
School type	Government	205 (84.36)	179 (73.66)	0.005
	Private	11 (4.53)	9 (3.70)	
	Never admitted	27 (11.11)	54 (22.22)	
Height-for-age	Normal	111 (45.68)	124 (51.03)	0.008
	Mild stunting	87 (35.80)	55 (22.63)	
	Moderate stunting	16 (6.58)	28 (11.52)	
	Severe stunting	29 (11.93)	36 (14.81)	
BMI-for-age	Normal	169 (69.55)	150 (61.73)	0.193
	Moderate thinness	35 (14.40)	44 (18.11)	
	Severe thinness	39 (16.05)	49 (20.16)	

To assess gender as the exposure rather than as an outcome, binomial logistic regression was performed with each adverse educational or nutritional outcome as the dependent variable and gender (female vs. male) as the predictor (Table 3). Females had significantly higher odds of never being enrolled (OR: 2.29; 95% CI: 1.38-3.77; p=0.001) and of having moderate or severe stunting (OR: 1.57; 95% CI: 1.02-2.42; p=0.040). The odds of moderate or severe thinness were higher among females but did not reach statistical significance (OR: 1.42; 95% CI: 0.97-2.06; p=0.070), consistent with the non-significant chi-square result for BMI-for-age. Overall, the findings demonstrate a gender disparity in educational access and in chronic undernutrition (stunting), with females disadvantaged on both, alongside a non-significant trend toward greater thinness among females.

**Table 3:** Odds of adverse educational and nutritional outcomes among girls relative to boys. Binomial logistic regression with gender (female vs. male) as the exposure. A p<0.05 was considered statistically significant.

Outcome (dependent variables)	Male, n/N (%)	Female, n/N (%)	OR (95% CI)	p-Value
Never enrolled	27/243 (11.11)	54/243 (22.22)	2.29 (1.38-3.77)	0.001
Moderate/severe stunting	45/243 (18.52)	64/243 (26.34)	1.57 (1.02-2.42)	0.040
Moderate/severe thinness	74/243 (30.45)	93/243 (38.27)	1.42 (0.97-2.06)	0.070

## Discussion

This community-based study of 486 children aged seven to 12 years in Jasra block, Prayagraj, with equal representation of boys and girls, found that gender disparity was multidimensional but indicator-specific. Girls were disadvantaged in educational access (never enrolment 22.22% vs. 11.11%; p=0.001) and in chronic undernutrition, with significantly higher moderate and severe stunting (p=0.008). BMI-for-age-based thinness was proportionately higher among girls but was not statistically significant (p=0.193) and is therefore best regarded as an observed trend. The regression analysis, with gender treated as the exposure, reinforced this pattern: girls had significantly higher odds of never being enrolled and of stunting, but not of thinness.

Educational disparity

The educational disadvantage of girls is central to interpreting these findings, because school participation is itself a platform for health and nutrition services such as mid-day meals, deworming, iron-folic acid supplementation, and periodic health checks. A girl who is never enrolled is excluded not only from education but also from these routine delivery channels, so educational exclusion can amplify nutritional risk. This is consistent with evidence that educational delay and exclusion coexist with poor nutritional status in disadvantaged Indian populations and with local findings from Prayagraj of school dropout, illiteracy among mothers, and poor dietary practices among adolescent girls [1,8]. The present results suggest that this disadvantage begins before adolescence, in the seven to 12-year age group.

Nutritional disparity

The higher burden of stunting among girls is consistent with several Indian studies. Tripathi et al., working in the same rural block, reported higher severe underweight and mild-to-moderate stunting among girls [4]; Pal and Gautam in Varanasi reported higher severe malnutrition among girls and a marked excess in government-school children [6]; and broader district and state estimates confirm a high background burden of stunting and underweight in this region [7,10]. Patel et al., studying children under five years of age in an urban slum in Western Maharashtra, similarly found higher rates of grade II-III malnutrition and poorer height-for-age among girls [11], and Kaur et al., among adolescents in Uttarkashi, reported higher rates of moderate stunting among girls [12]. At the same time, the direction of gender disparity is not uniform across India. Jawaregowda and Angadi reported higher undernutrition among boys in Karnataka [3], a national sibling-pair analysis found little intra-household difference [13], a nationally representative analysis of mother-child pairs underscored the multidimensional, socially patterned nature of malnutrition in India [14], and an infant study from Ethiopia found boys more affected [15]. These contrasts indicate that gender disparity in nutrition is context-specific and indicator-specific; the present findings should be read as evidence of local female disadvantage in selected indicators, not as a universal claim that girls are always more malnourished. Accordingly, the non-significant difference in thinness is interpreted as a trend rather than a confirmed disparity.

Where female disadvantage is observed, social rather than biological mechanisms are the most plausible explanation. Intra-household food allocation that disfavors girls [2], lower nutrient intake among females reported elsewhere [16], and gendered caregiving expectations and food sacrifice documented in South Asian settings can plausibly produce greater chronic deficit (stunting) and a tendency toward thinness among girls [17]. Maternal education, caste, family type, and household income further modify these relationships [5]. The coexistence of educational and nutritional disadvantages among girls in this community is consistent with such household-level and normative processes, although dietary intake was not measured here, and these pathways could not be tested directly.

These findings call for integrated rather than isolated action. Community-level nutrition screening of school-age children should be strengthened, with particular attention to girls who are never enrolled, irregular, or have dropped out, since they are least likely to be reached through school-based services. United Nations Children's Fund (UNICEF) has emphasized that school-age children represent a critical period during which lifelong dietary habits are shaped and that hidden hunger can impair attention and learning, reinforcing the importance of reaching this age group [18]. Anganwadi workers, Accredited Social Health Activists (ASHAs), school teachers, and local government officials should jointly identify and link vulnerable children to services. Nutrition education should emphasize equitable intra-household food allocation, dietary diversity using affordable local foods, and regular protein-containing meals. Because school type and enrolment were associated with gender, school-retention and nutrition interventions are best planned together, with a gender-sensitive approach to prevent school-age deficits from carrying into adolescence and future motherhood.

Strengths and limitations

Strengths include the community-based design, which captured children outside the school system; a relatively large sample (486) with equal gender representation; a focus on the under-studied seven to 12-year age group; simultaneous assessment of educational and nutritional indicators; and direct anthropometric measurement. Several limitations should be noted. First, the cross-sectional design cannot establish causality. Second, the study was confined to a single rural block, so the findings should not be generalized to the wider district or state without caution. Third, the consecutive, non-probability sampling approach, although implemented as near-complete house-to-house enrolment of eligible children within the block, may still introduce selection bias and limit representativeness. Fourth, educational and school-status information was caregiver-reported and subject to recall or reporting bias. Fifth, several factors known to influence both nutritional and educational outcomes - parental education, dietary intake, household food security, and child morbidity - were not measured and therefore could not be adjusted for, which may confound the observed gender associations.

## Conclusions

Among children aged seven to 12 years in Jasra block, gender disparity was evident in educational access and in chronic undernutrition. Girls had significantly higher never enrolment and a greater burden of moderate and severe stunting, and educational status, school type, and height-for-age were significantly associated with gender. BMI-for-age-based thinness was higher among girls, but did not reach statistical significance and should be interpreted as a non-significant trend. Nutritional disadvantage among girls cannot be addressed through food supplementation alone; it is linked with educational access, school retention, household practices, and gender norms. Community-based nutrition screening, gender-sensitive school health services, active tracking of never-enrolled and out-of-school girls, and household counseling on equitable food distribution are needed to prevent school-age deficits from persisting into adolescence and future motherhood.
